# Highlight report: Predicting late metastasis in breast cancer

**DOI:** 10.17179/excli2016-843

**Published:** 2016-12-23

**Authors:** Seddik Hammad, Gada S. Osman, Mohamed Ezzeldien, Hassan Ahmed, Ahmed M. Kotb

**Affiliations:** 1 Department of Forensic Medicine and Toxicology, Faculty of Veterinary Medicine, South Valley University, Qena, Egypt; 2 Molecular Hepatology - Alcohol Associated Diseases, Department of Medicine II, Medical Faculty Mannheim, University of Heidelberg, Mannheim, Germany; 3 Department of Pathology, Faculty of Medicine, South Valley University, Qena, Egypt; 4 Department of Physics, Faculty of Science, South Valley University, Qena, Egypt; 5 Department of Physiology, Faculty of Veterinary Medicine, South Valley University, Qena, Egypt; 6 Institute of Anatomy and Cell Biology, University Medicine Greifswald, Greifswald, Germany; 7 Department of Anatomy and Histology, Faculty of Veterinary Medicine, Assiut University, Assiut, Egypt

## ⁯

Recently, Birte Hellwig and colleagues from the Department of Statistics, TU Dortmund University have published a study to predict late metastasis in breast cancer (Hellwig et al., 2016[8]). In breast cancer survival strongly depends on distant metastasis (Schmidt et al., 2008[17]). Although, the risk of metastasis decreases with time after surgery, metastatic events do still occur more than five years after diagnosis (Karrison et al., 1999[9]; Demicheli et al., 1996[2]; Saphner et al., 1996[16]). Prediction of these late metastatic events is of high relevance. On the one side, the patient's distress would be alleviated if a low probability of late metastatic recurrence could be predicted (Hellwig et al., 2016[8]). On the other hand, high-risk patients could be integrated into extended endocrine therapy studies. 

In the present study Hellwig et al. (2016[8]) used a sequential validation strategy to identify and validate genes that predict late metastasis: they identified a first set of late genes in a breast cancer cohort (n=409), which was confirmed in an independent validation cohort (n=169) and additionally confirmed in a second validation cohort (n=923). The careful sequential validation that also includes a check for sample annotation errors (Grinberg et al., 2015[5]) is certainly strength of the present study. The authors validated nine late-type genes, whereby the tumor angiogenesis modifier EPN3 was associated with increased and several ribosome-related genes with decreased risk of late breast cancer metastasis. 

In recent years numerous studies have been performed to predict prognosis of cancer (Hammad, 2013[6]; Hammad et al., 2013[7]; Marchan, 2014[13][12]; Lohr et al., 2015[11]). Often, prognostic factors are associated with proliferation (Schmidt et al., 2008[17], 2012[18]), immune cell infiltration (Lohr et al., 2013[10]; Godoy et al., 2014[4]), inflammation (Mattson et al., 2015[14]; Sicking et al., 2014[19]), tumor cell migration (Stock et al., 2015[21]; Stewart et al., 2012[20]), disturbed circadian control (Cadenas et al., 2014[1]; Ghallab, 2015[3]) or antioxidant status (Milicevic et al., 2014[15]). However, most previous studies did not differentiate, whether prognostic factors predict early or late metastasis. The present study of Hellwig and colleagues underlines that the vast majority of all prognostic genes in breast cancer predicts metastasis only up to approximately three years after surgery. Only a small minority of prognostic genes maintains its significance at later periods. The relevance of the present study is that these 'late genes' have so far not been used for routine diagnosis of breast cancer recurrence risk and may lead to an improvement of the accuracy of already existing systems.
